# Impairment of Skeletal Muscle Contraction by Inhibitors of GABA Transporters

**DOI:** 10.3390/ijms252312510

**Published:** 2024-11-21

**Authors:** Nikita S. Fedorov, Guzel V. Sibgatullina, Artem I. Malomouzh

**Affiliations:** 1 Kazan Institute of Biochemistry and Biophysics, Federal Research Center “Kazan Scientific Center of RAS”, 2/31 Lobachevsky Street, P.O. Box 30, Kazan 420111, Russia; trane.ask@mail.ru (N.S.F.); kam-guz@yandex.ru (G.V.S.); 2 Department of Radiophotonics and Microwave Technologies, Kazan National Research Technical University, 10 K. Marx St., Kazan 420111, Russia

**Keywords:** neuromuscular junction, skeletal muscle, acetylcholine, GABA, GABA transporter, GABA_B_ receptor, muscle contraction

## Abstract

γ-Aminobutyric acid (GABA) has a significant impact on the functioning of not only the central but also the peripheral part of the nervous system. Recently, various elements of the GABAergic signaling system have been discovered in the area of the neuromuscular junction of mammals. At the same time, the functional activity of membrane-bound GABA transporters (GATs) and their role in neuromuscular transmission have not been identified. In the present study, performed on a neuromuscular preparation of the mouse diaphragm, the effect of GABA transporter inhibitors (nipecotic acid and β-alanine) on the force of muscle contraction was assessed. It was found that in the presence of both compounds in the bathing solution, the force of contractions caused by stimulation of the motor nerve dropped by 30–50%. However, when the muscle was stimulated directly, no effect of GABA transporter inhibitors on the contractile force was observed. The depressant effect of β-alanine induced by nerve stimulation was completely abolished by the GABA_B_ receptor blocker CGP 55845. GABA transporters were detected at the neuromuscular junction using immunohistochemistry. Thus, our results indicate that GABA transporters are localized in the area of the neuromuscular junction, and their activity affects the muscle contraction force. This influence is most likely due to the removal of GABA released during nerve stimulation and activating GABA receptors, which leads to a decrease in the contraction force of the striated muscles.

## 1. Introduction

γ-Aminobutyric acid (GABA) is considered to be the main inhibitory neurotransmitter in CNS synapses. It is involved in the development, maturation, and functioning of the nerve tissue [1,2,3,4,5]. The intercellular signaling function of the amino acid is mediated by the activation of ionotropic GABA_A_ [6,7] and metabotropic GABA_B_ receptors [8,9], which can have both pre- and postsynaptic localization. The implementation of the GABA signaling function largely depends not only on the activation of receptors but also on the activity of membrane-bound GABA transporters (GATs), which rapidly remove amino acids from the extracellular space and, thereby, terminate synaptic signaling. To date, six proteins have been identified that are capable of transporting GABA molecules across the plasma membrane [10], of which four proteins (GAT-1, GAT-2, GAT-3, and the betain/GABA transporter type 1) are most often considered in intercellular signaling [11]. Moreover, GAT-1 and GAT-3 are the two main subtypes of GATs responsible for the regulation of extracellular GABA levels in the central nervous system [12].

A number of studies performed on various parts of the peripheral nervous system have obtained data indicating the signaling (modulatory) synaptic function of GABA outside the CNS [13,14]. Thus, in particular, in the area of the neuromuscular synapse of rats, glutamate decarboxylase, the enzyme involved in the synthesis of GABA, molecules of GABA, GABA_B_ receptors and also GAT-2, was detected [15,16]. Activation of GABA_B_ receptors by an exogenous agonist leads to a decrease in the release of acetylcholine from motor nerve endings [17]. This, in turn, can lead to an impairment in neuromuscular transmission and, as a consequence, a decrease in the contraction force of the striated muscles. This assumption was confirmed in a study that demonstrated an increase in the contraction force of striated mouse muscle with indirect stimulation after the application of GABA receptor blockers [18]. Thus, the data obtained to date suggest an endogenous release of GABA during stimulation of the motor nerve and the existence of a modulatory function of the amino acid, affecting the contractile activity of muscle. However, no data on the mechanisms regulating this function via GABA transporters in the neuromuscular junction have been obtained to date. This determined the necessity for the present study, which was decided to be carried out on the same neuromuscular preparation of the mouse diaphragm, on which data about the effect of GABA receptors on muscle contractions were obtained [18].

The main aim of the present study was to evaluate the effects of GAT blockers on the force of striated muscle contractions induced by indirect and direct stimulation. In addition, for experiments on the neuromuscular preparation of the mouse, an important task was to obtain immunohistochemical evidence of the presence of GABA transporters, as was performed previously on the rat neuromuscular junction [16].

## 2. Results

### 2.1. Contractile Activity of the Diaphragm in Control

**Figure 1:**
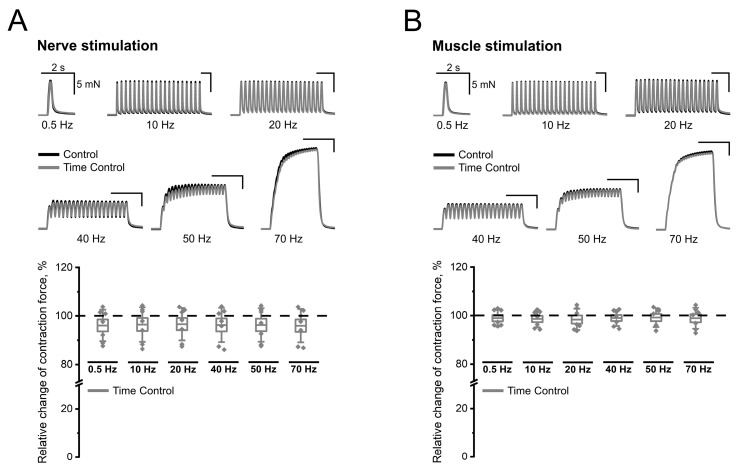
Twitches and tetanic contractions of the mouse diaphragm at different frequencies of indirect (nerve; panel (**A**)) and direct (muscle; panel (**B**)) stimulation at the beginning (control) and at the end (time control) of the experimental protocol. Traces of contractions from representative control experiment (upper parts of panels) are shown. Data (lower parts of panels) are presented as box plots with data overlap: diamonds—relative changes in individual experiments (n = 7 mice); horizontal lines—M ± SEM (boxes) and SD (whiskers). For all data groups, the *p* value is greater than 0.05 (two-tailed *t*-test).

Since the contraction force may decrease when conducting an experiment at an ambient temperature above 30 °C [19,20], the first stage of this study was to establish to what extent the force of contractions changes when using our protocol. The average values of the contraction force of the diaphragm fragments were 7.34 ± 1.05 mN and 5.84 ± 1.32 mN (at 0.5 Hz), 7.16 ± 1.06 mN and 6.01 ± 1.40 mN (at 10 Hz), 7.27 ± 1.07 mN and 5.98 ± 1.42 mN (at 20 Hz), 11.07 ± 1.76 mN and 9.13 ± 1.69 mN (at 40 Hz), 18.18 ± 2.82 mN and 15.03 ± 2.39 mN (at 50 Hz), and 33.05 ± 4.01 mN and 28.41 ± 3.46 mN (at 70 Hz), with indirect and direct muscle stimulation, respectively. During the protocol, no significant change in the measured parameter were noted, although the average value of the contraction force was lower in the first case by 4–5% (Figure 1A), while in the second case it was only 1–2% (Figure 1B).

Thus, when using our research protocol, no reliably significant changes in the contraction force were observed at any of the stimulation frequencies used (both with indirect and direct modes of inducing contraction).

### 2.2. Effect of Nipecotic Acid on Contractile Activity of the Diaphragm

One of the widely used GABA transporter inhibitors is nipecotic acid [21,22,23]. To completely inactivate GABA uptake in brain preparations, this compound is used in the millimolar range. In particular, in the works [24,25], nipecotic acid was used at a concentration of 10 mM. In this regard, we also opted for this concentration.

**Figure 2:**
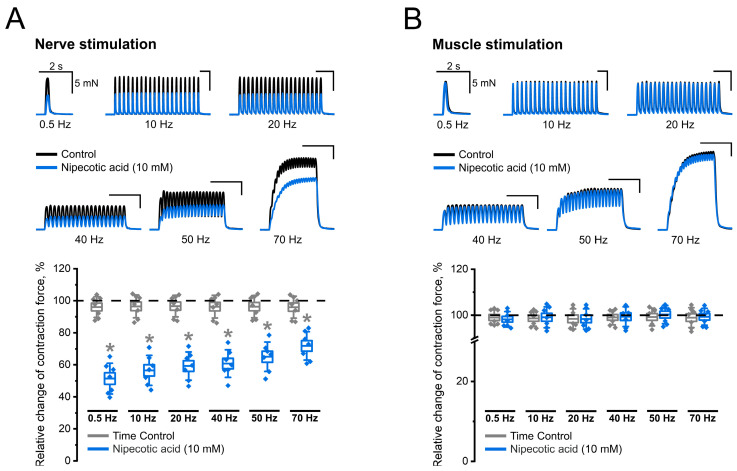
Inhibitory effect of nipecotic acid (10 mM) on contractions caused by indirect (nerve) stimulation (panel (**A**)) and complete absence of its effect on contractions caused by direct (muscle) stimulation (panel (**B**)). The upper parts of the panels show native records of contractions obtained in individual experiments in control (black) and 20 min after nipecotic acid application (blue). The lower parts of the panels show the relative changes in the contraction force after nipecotic acid application relative to control (%) and time control. Data are presented as box plots with data overlap: diamonds—relative changes in individual experiments (n = 7 mice); horizontal lines—M ± SEM (boxes) and SD (whiskers). * *p* < 0.05 vs. time control (Mann–Whitney U test).

The application of nipecotic acid in the above concentration resulted in a marked decrease in the force of the recorded contractions caused by stimulation of the motor nerve at all frequencies used (Figure 2A). The decrease in the contraction amplitude was from 27 to 50%. At the same time, no effect of the GABA transporter inhibitor on the force of contractions caused by direct stimulation was detected (Figure 2B). The average values of the amplitudes of the recorded responses were completely consistent with the values obtained both before the application of the pharmacological agent and with the values of the time control.

Thus, nipecotic acid negatively affects the contractile activity of skeletal muscle caused by nerve stimulation. To confirm that this is due to the ability of the pharmacological agent to inhibit the GABA uptake system, the following series of experiments were performed using the same protocol but with another classical inhibitor of GABA transporters—β-alanine [26].

### 2.3. Effect of β-Alanine on Contractile Activity of the Diaphragm

Despite the structural differences in the molecular structure of nipecotic acid and β-alanine [26], the latter is also used in physiological experiments in the millimolar concentration range to inactivate GABA transport by various amino acid transporters. Based on the literature [27,28], we chose a concentration of 1 mM.

**Figure 3:**
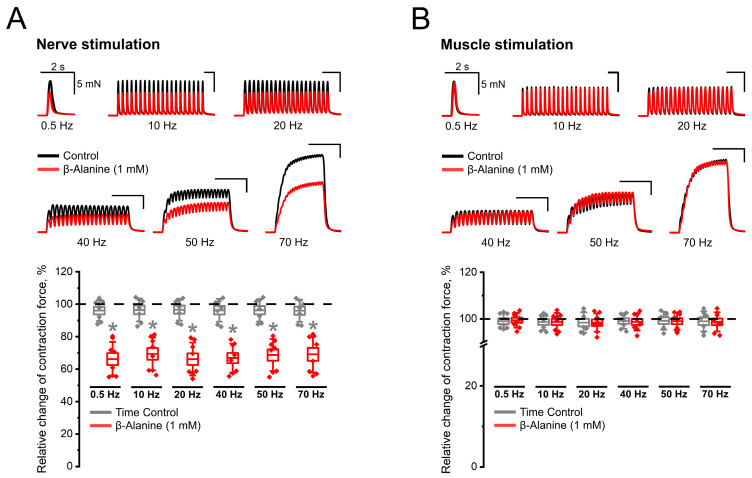
Inhibitory effect of β-alanine (1 mM) on contractions caused by indirect (nerve) stimulation (panel (**A**)) and complete absence of its effect on contractions caused by direct (muscle) stimulation (panel (**B**)). The upper parts of the panels show native records of contractions obtained in individual experiments in control (black) and 20 min after β-alanine application (red). The lower parts of the panels show the relative changes in the contraction force after β-alanine application relative to control (%) and time control. Data are presented as box plots with data overlap: diamonds—relative changes in individual experiments (n = 8 mice); horizontal lines—M ± SEM (boxes) and SD (whiskers). * *p* < 0.05 vs. time control (Mann–Whitney U test).

The addition of β-alanine to the Ringer–Krebs solution resulted in a contraction force caused by nerve stimulation falling by 30–35% both at a low and high frequency of stimulation (Figure 3A). At the same time, the amplitude of contractions caused by direct muscle stimulation, as in the previous series of experiments, did not undergo any changes (Figure 3B). The contraction force values were completely consistent with the control level.

Thus, as in the case of nipecotic acid, β-alanine negatively affects only contractions caused by nerve stimulation. In other words, both pharmacological agents used by us, capable of inhibiting the uptake of GABA into cells, do not affect the process of muscle contraction itself but unidirectionally reduce contractile activity initiated by synaptic transmission at the neuromuscular junction. And if the effects of the studied agents are mediated by the accumulation of GABA in the synapse, then the blockade of GABA receptors should eliminate the effect of inhibition of transporters.

### 2.4. Elimination of the Effect of β-Alanine on Nerve-Stimulated Muscle Contractions by a GABA_B_ Receptor Blocker

Since metabotropic receptors for GABA (GABA_B_ receptors) were identified in the neuromuscular junction [15,16] and their pharmacological blockade eliminated the inhibitory effect of exogenous GABA on the process of acetylcholine release from the motor nerve terminal [17], it is likely that these proteins can mediate the effects of GABA, the molecules of which accumulate in the synapse when transporters are blocked.

**Figure 4:**
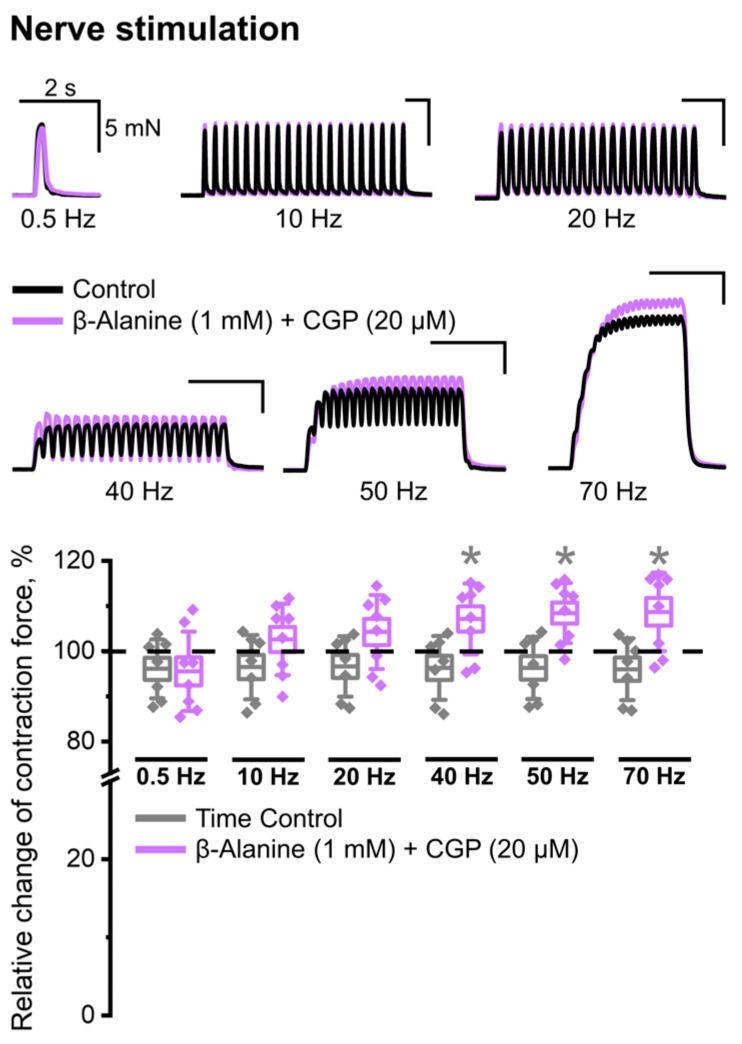
The absence of an inhibitory effect of β-alanine (1 mM) on contractions caused by indirect stimulation in the presence of a GABA_B_ receptor blocker CGP 55845 (20 µM) in the Ringer–Krebs solution. The upper parts of the panels show native records of contractions obtained in individual experiments in control (black) and 20 min after chemical application (violet). The lower parts of the panels show the relative changes in the contraction force after β-alanine and CGP 55845 application relative to control (%) and time control. Data are presented as box plots with data overlap: diamonds—relative changes in individual experiments (n = 8 mice); horizontal lines—M ± SEM (boxes) and SD (whiskers). * *p* < 0.05 vs. time control (Mann–Whitney U test).

In the presence of the potent and selective GABA_B_ receptor antagonist CGP 55845 (20 μM) in solution, no inhibitory effect of β-alanine (1 mM) was detected at any of the motor nerve stimulation frequencies used (Figure 4). Moreover, the amplitude of contractions evoked by stimulation at 40, 50, and 70 Hz was even higher than the control values.

The complete elimination of the β-alanine effect by the GABA_B_ receptor blocker confirms the fact that it is due to the inhibition of GABA transporters that amino acid molecules accumulate in the synaptic cleft, which has an inhibitory effect on nerve-induced muscle contractions. In other words, we have obtained pharmacological evidence of the presence and functioning of GABA transporters, the activity of which is manifested during nerve stimulation. Consequently, these proteins should be localized in the area of synaptic contact. To test this assumption, an immunohistochemical study was conducted to identify the localization of GABA transporters in the neuromuscular preparation of a mouse.

### 2.5. Immunolocalization of GABA Transporters in Neuromuscular Preparation of Mouse Diaphragm

To date, GABA transporters have been detected immunohistochemically only in the rat diaphragm [16]. To assess not only the localization but also, possibly, the profile of these proteins, antibodies to the following three transporters were used: GAT-1, GAT-2, and GAT-3. To visualize the synaptic contact zone, a “classical” approach was used—staining of postsynaptic cholinergic receptors by TMR-α-bungarotoxin.

**Figure 5:**
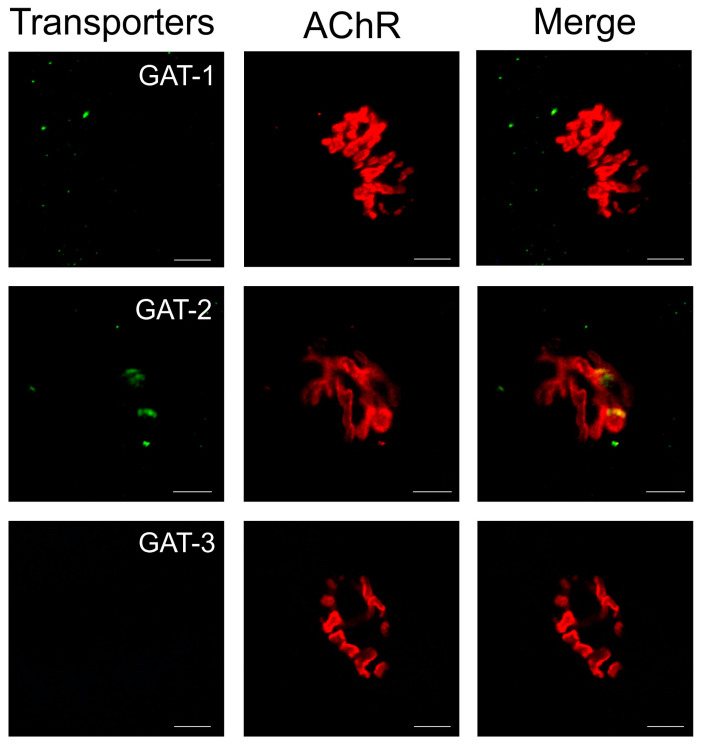
Micrographs from mouse diaphragm neuromuscular preparations processed for immunohistochemical analysis with specific antibodies against GABA transporters (GAT-1, GAT-2, and GAT-3; green). Labeling of nicotinic acetylcholine receptors (AChR) with tetramethylrhodamine-α-bungarotoxin was used as a marker of postsynaptic membrane of skeletal muscle fibers and synaptic region of neuromuscular preparation (red). Weak immunopositive reaction was revealed with antibodies against GAT-1; more pronounced immunopositive staining was detected when using antibodies against GAT-2. At the same time, immunohistochemical reaction with antibodies against GAT-3 was not detected. Scale bar = 10 μm.

When using antibodies to GAT-1, barely noticeable immunohistochemical staining was detected in the form of small dots near the end plate zone (Figure 5). A more pronounced immunohistochemical reaction was detected in the synaptic contact zone when using antibodies to GAT-2. However, no staining was observed in any of the preparations where antibodies to GAT-3 were used (Figure 5).

Thus, the detection of specific immunohistochemical staining of GABA transporters in the preparations we studied confirms the presence of these proteins in the region of the peripheral cholinergic synapse.

## 3. Discussion

In this work, experimental evidence is obtained for the first time that the functional activity of GABA transporters is detected in the mammalian neuromuscular synapse, which affects muscle contractility. Inhibition of these proteins reduces the force of muscle contractions caused by stimulation of the motor nerve, and this effect does not occur in the presence of a GABA_B_ receptor blocker.

The discovery of the therapeutic potential of GABA receptor activation in alleviating muscle spasticity [29] prompted research into the possible role of GABA in the functioning of the peripheral nervous system. Interestingly, binding sites characteristic of the benzodiazepine site of GABA_A_ receptors were found on the rat diaphragm preparation [30]. Antagonists of this site increased muscle contractility at both direct and indirect stimulation of the muscle, did not affect either spontaneous or evoked quantal release of acetylcholine, and did not change the sensitivity of the muscle membrane to neurotransmitter [31]. Relatively recently, using immunohistochemistry, we identified GABA_B_ receptors in the neuromuscular synapses of the diaphragm [17], as well as in the synapses of the “fast” (m. EDL) and “slow” (m. soleus) skeletal muscles of the rat [15]. The specific immunohistochemical staining pattern indicated a presynaptic localization of the receptors. Electrophysiological data support this assumption since the application of exogenous GABA caused a decrease in the level of acetylcholine release, both tonic and induced by electrical stimulation of the nerve [17]. It should be noted that the inhibitory effect of presynaptic GABA receptors on the process of acetylcholine release from nerve endings was previously noted in a number of other preparations from the peripheral nervous system of mammals [32,33,34]. Moreover, a decrease in smooth muscle contractile activity has been demonstrated by the activation of GABA_B_ receptors [32,33]. A similar effect was observed in striated muscle, where receptor blockade increased contractions induced by nerve stimulation [18]. The results of this study suggest that the GABA receptor agonist released during nerve stimulation reduces the force of muscle contractions, most likely by reducing the amount of acetylcholine secreted [17]. Therefore, if GABA is released in the neuromuscular junction, then blockade of GABA transporters should lead to a marked decrease in the force of contractions since, in this case, the amino acid will accumulate in the synaptic cleft. This is exactly the effect we observed in our study using nipecotic acid and β-alanine. And the data from a series of experiments with direct stimulation and with a GABA_B_ receptor blocker confirm that the observed effects of nipecotic acid and β-alanine (i) are not associated with the influence of the compounds directly on the muscle, (ii) are realized only during stimulation of the motor nerve, and (iii) involve GABA_B_ receptors. Thus, we have obtained further evidence of endogenous release of GABA in the neuromuscular junction caused by stimulation of the motor nerve.

Where and how can GABA be released into the synaptic cleft of a peripheral cholinergic synapse? According to modern concepts, the neuromuscular junction (like any synaptic contact) should be considered from the position of a tripartite synapse, i.e., this is an intercellular contact where direct interaction of the motor neuron, muscle fiber, and terminal Schwann cells surrounding this contact occurs [35,36,37]. At the moment, it is difficult to give an unambiguous answer to this question since all three compartments of the neuromuscular junction can act as a source of GABA in the synaptic cleft.

*Muscle fiber as a source of GABA.* Some researchers suggest that one of the potential sources of amino acid release into the extracellular space may be skeletal muscle fiber. So, a certain amount of GABA was detected during metabolomic analysis of the skeletal muscle of mice [38]. The revealed correlation of the increase in GABA in muscle and plasma due to long-term exercise training also suggests that GABA can act as a myokine-like molecule [39]. At the same time, during the immunohistochemical analysis of the rat neuromuscular preparation, GABA was detected exclusively in the area of the synaptic contact, and there is reason to believe that the amino acid has a presynaptic, not muscle localization [16]. The presence of amino acids in nerve endings located in the structure of skeletal muscle may affect the biochemical assessment of GABA levels in muscle tissue. This can be completely avoided by using muscle cell culture. Indeed, under these conditions, GABA is identified in myotubes [39], but it is apparently synthesized in relatively large quantities in developing elements of muscle tissue. So, using the same antibodies to GABA as in the work [16], where the amino acid was not detected in mature muscle, strong immunohistochemical staining for GABA is detected in the culture of rat myocytes and myotubes [40]. It is interesting to note that this staining, although less pronounced, is also detected in the muscle tissue of newborn animals, whereas in the muscle of an adult organism, GABA again ceases to be detected immunohistochemically. In this regard, an assumption was made about the participation of GABA in the processes of muscle tissue development in the early stages of ontogenesis [41].

*Glial cells as a source of GABA.* The direct involvement of glial cells in the functioning of synaptic contact and, in particular, in the processes of synaptic plasticity is already an established fact [42,43], including in the neuromuscular synapse [44,45]. Glial cells are capable of releasing synaptically active molecules called gliotransmitters in response to neuronal activity, and GABA is considered the main gliotransmitter [46,47].

*Motor neuron as a source of GABA.* At present, however, there are still most prerequisites for the fact that GABA can be released from the presynaptic nerve terminal as a co-transmitter of acetylcholine [14]. The cooperative release of these two neurotransmitters from cholinergic neurons in the central nervous system is already an established fact [48,49,50,51]. In this regard, it is necessary to note the recent study by Castagnola et al. [52], which provided further evidence not only of the cotransmission of acetylcholine and GABA from cholinergic nerve terminals but also of the GABA_B_ receptor-mediated inhibitory effect of the amino acid on the acetylcholine release.

In any case, when stimulating the motor nerve, we observe a decrease in the contraction force in the presence of pharmacological agents that inhibit the GABA uptake process. To expand the evidence base that this effect is mediated by amino acid transporter proteins, we attempted to use immunohistochemistry to determine the localization of GABA transporters.

Despite the use of antibodies specific to certain subtypes of GABA transporters, it is still premature to state which subtypes of transporters are present or absent. This is due to several reasons. Firstly, we used antibodies for only three transport proteins that are usually considered in terms of GABAergic signaling [11], while this function can be performed by at least six proteins [10]. Secondly, the nomenclature of GABA transporters in mice is different and somewhat confusing when compared to the nomenclature of GABA transporters in rats. So, in mice, GAT-1 carries the same name as in rats, while GAT-2 corresponds to BGT1 and GAT-3 corresponds to GAT-2 [10]. Thirdly, we used classical GABA transporter inhibitors at concentrations that do not indicate a specific blockade of any one type of protein [23,24,25,27,28]. Nevertheless, the use of different antibodies in the present study allows us not only to state that transporter proteins are indeed localized mostly near the synaptic region but also that these proteins are represented by at least two types. To establish the subtypes of transporters expressed in the neuromuscular preparation, it is necessary to perform a detailed investigation using biochemical and molecular genetic methods.

Overall, the obtained results confirm our data about the presence and functioning of the GABAergic signaling pathway in the peripheral cholinergic synapse [15,16], which is capable of modulating the processes of neuromuscular transmission [17] and, as a result, regulating the contractile activity of skeletal muscles [18]. At the same time, the data of the present study prove the significance of the role of GABA transporters in the implementation of this regulatory pathway. Thus, a foundation is being laid for the development of new strategies and methods for correcting neuromuscular dysfunctions by influencing GABA transporters, the therapeutic potential of which is currently being actively studied in the context of a number of diseases [53,54,55,56].

## 4. Materials and Methods

**Animals.** Mice BALB/C (2–3 months old, weighing 20–25 g) of both sexes were used in this study. Animals were kept in sawdust-lined plastic cages in a well-ventilated room with a 12 h light/dark cycle and given ad libitum access to food and water. All animal care and experimental protocols were approved by the Bioethics Committee of Kazan Federal Scientific Centre. All efforts were made to minimize animal suffering.

**Tissue preparation and bathing solution.** Experiments were performed on neuromuscular preparations from diaphragms excised from mice. Animals were euthanized by cervical dislocation in accordance with the approved project protocol. Hemidiaphragms with their associated phrenic nerves were isolated from the mice and mounted in a temperature-controlled chamber filled with an oxygenated Ringer–Krebs solution of the following composition (mM): NaCl (135), KCl (5), CaCl_2_ (2), MgCl_2_ (1), NaH_2_PO_4_ (1), NaHCO_3_ (11.9), and glucose (11). The pH was maintained at 7.3–7.4 by bubbling with 95% O_2_ and 5% CO_2_. Then, the hemidiaphragms were cut lengthwise into strips of intact muscle fibers (the width of the muscle fragment was 7–9 mm) and were used in the experiments.

**Twitch tension measurements.** The experimental protocol for direct and indirect twitch tension measurements was similar to the one described in [18,57] with some modifications. Muscle contractions were recorded using an SIH Muscle Tester (WPI, Sarasota, FL, USA) equipped with an SI-BAM21-LC amplifier (WPI) and an SI-TCM2 temperature controller (WPI).

To measure the force, muscles were mounted between a force transducer (SI-KG2B, WPI) and a fixed hook. For each preparation, the resting tension was adjusted at the beginning of the experiment (to obtain maximal contractile response). In addition, before the start of each experiment, the preparation was washed with a Ringer–Krebs solution under conditions of rare stimulation of the motor nerve (1 imp/min, suprathreshold amplitude and duration of 0.1 ms) to achieve a “steady state” (on average, this lasted 15–25 min).

Responses to indirect stimulation were evoked by stimulating the phrenic nerve via a suction electrode. During direct stimulation, diaphragm contractions were evoked by stimulation via two silver wire electrodes located in the experimental bath in close proximity to the preparation. The stimulation protocol (both for direct and indirect stimulation) was identical: 10 stimuli at 0.5 Hz, 20 stimuli at 10 Hz, 20 stimuli at 20 Hz, 20 stimuli at 40 Hz, 20 stimuli at 50 Hz, and 20 stimuli at 70 Hz. The time between stimulation periods was 10 s.

The protocol of the experiment for recording contractility was as follows: control recording of contractile responses during nerve stimulation and after 2–3 min during direct muscle stimulation, then recording of responses (during direct and indirect stimulation) after 15–20 min after application of pharmacological agents. Experiments were performed at 32.0 ± 0.2 °C.

This study used the classical inhibitors of neuronal and astrocytic GABA transport nipecotic acid and β-alanine [21], as well as the GABA_B_ receptor antagonist CGP 55845 ((2S)-3-[[(1S)-1-(3,4-Dichlorophenyl)ethyl]amino-2-hydroxypropyl](phenylmethyl)phosphinic acid hydrochloride). Chemicals were purchased from Sigma-Aldrich, St. Louis, MO, USA.

Signals from the force sensor were acquired and analyzed digitally, using LabTrax 8/16 system (WPI) and “MDAC 64-bit Software version 3.1.2”. The force of the contractions was measured in Newtons and then converted into percentages compared to control values.

**Immunohistochemistry.** The following primary antibodies were used in this study: GAT-1 GABA Transporter 1 (AGT-001, Alamone Labs, Jerusalem, Israel), GAT-2 GABA Transporter (AGT-002, Alamone Labs), and GABA T-3 Antibody (R-19) (sc-7669, Santa Cruz Biotechnology, Dallas, TX, USA).

Fixation of neuromuscular preparations was performed in a 4% paraformaldehyde solution. Samples were washed in phosphate buffer solution (PBS) for 30 min, changing the solution every 10 min. Then, they were incubated in 0.5% Triton X 100 solution (Sigma) in PBS for 40 min. Then incubated for 15 min in a blocking solution of the following composition: 1% bovine serum albumin BSA, 5% donkey serum and 0.5% Triton X-100. Then the samples were placed in a solution with antibodies to GABA transporters (1:200) for 15 h at 4 °C. The samples were then washed in 0.5% Triton X 100 and incubated for 1 h in a solution containing secondary antibodies conjugated with Alexa 488 (for GAT-1 and GAT-2), Alexa 647 (for GAT-3) (1:100, Invitrogen, Carlsbad, CA, USA), and tetramethylrhodamine-α-bungarotoxin (Btx) (Sigma, 1:50). After washing, the preparations were embedded in Sub-X resin (Leica, Wetzlar, Germany).

The preparations were examined using a Leica TCS SP5 MP confocal laser scanning microscope (Leica).

**Statistics.** Data were analyzed using “OriginPro 2021b (64-bit) SR2 version 9.8.5.212” software. Results in the text are shown as Mean ± SE (n, the number of independent experiments on separate muscles from individual mice). Data were tested for normality (using the Shapiro–Wilk test) and variance homogeneity (using the two-sample F test for variance). Statistical significance of the difference between groups (* *p* < 0.05) was assessed using a two-tailed *t*-test (for parametric and paired data) and Mann–Whitney U test (for non-parametric data). There were no exclusions of outliers.

## Data Availability

The data sets supporting the results of this article are included within the article. Further inquiries can be directed to the corresponding author (A.I.M.).
